# Predictive models in emergency medicine and their missing data strategies: a systematic review

**DOI:** 10.1038/s41746-023-00770-6

**Published:** 2023-02-23

**Authors:** Emilien Arnaud, Mahmoud Elbattah, Christine Ammirati, Gilles Dequen, Daniel Aiham Ghazali

**Affiliations:** 1 Emergency Department, Amiens Picardy University Medical Center, Rond-Point Christian CABROL, F-80000 Amiens, France; 2 Laboratoire Modélisation, Information et Systèmes, University of Picardie Jules Verne, UR 4290, 33 rue Saint Leu, F-80039, Amiens, Cedex 1 France; 3 Faculty of Environment and Technology, University of the West of England, BS16 1QY Bristol, UK; 4 SimUSanté, Amiens Picardy University Medical Center, Rond-Point Christian CABROL, F-80000 Amiens, France; 5 INSERM UMR1137, “Infection, Antimicrobials, Modelling, Evolution”, University of Paris-Diderot, 16 rue Henri HUCHARD, F-75870, Paris, Cedex 18 France

**Keywords:** Preclinical research, Epidemiology

## Abstract

In the field of emergency medicine (EM), the use of decision support tools based on artificial intelligence has increased markedly in recent years. In some cases, data are omitted deliberately and thus constitute “data not purposely collected” (DNPC). This accepted information bias can be managed in various ways: dropping patients with missing data, imputing with the mean, or using automatic techniques (e.g., machine learning) to handle or impute the data. Here, we systematically reviewed the methods used to handle missing data in EM research. A systematic review was performed after searching PubMed with the query “(emergency medicine OR emergency service) AND (artificial intelligence OR machine learning)”. Seventy-two studies were included in the review. The trained models variously predicted diagnosis in 25 (35%) publications, mortality in 21 (29%) publications, and probability of admission in 21 (29%) publications. Eight publications (11%) predicted two outcomes. Only 15 (21%) publications described their missing data. DNPC constitute the “missing data” in EM machine learning studies. Although DNPC have been described more rigorously since 2020, the descriptions in the literature are not exhaustive, systematic or homogeneous. Imputation appears to be the best strategy but requires more time and computational resources. To increase the quality and the comparability of studies, we recommend inclusion of the TRIPOD checklist in each new publication, summarizing the machine learning process in an explicit methodological diagram, and always publishing the area under the receiver operating characteristics curve—even when it is not the primary outcome.

## Introduction

Predictive models are increasingly being used in the field of emergency medicine (EM), where they particularly deal with the prediction of triage, admission, and mortality^1^. A model is trained to predict an outcome variable (e.g., discharge status) as a function of covariables (e.g., vital signs or the reason for admission) that are also referred to as “predictors” or “features”. In the classical case of supervised machine learning (ML), the model is trained on a set of labeled data. In this context, the model’s performance depends directly on the dataset’s quality and exploitation.

In a supervised ML process, the dataset is split into a training set (used to predict the outcome on the basis of the predictors) and a test set (used to compare the algorithm’s prediction with the known outcome). The most usual train-test split ratio is 80:20. The model’s performance is measured by the area under the receiver operating characteristic curve (AUROC): a value of 0.5 corresponds to a random prediction, whereas a value of 1 corresponds to a perfect prediction. One of the risks associated with supervised ML is overfitting: the trained model is tightly correlated with the training set and will not accurately predict the outcome for new data. Hence, to ensure that the model’s performance is not overly dependent on the training set, external validation is required, this consists in computing the AUROC for an independent dataset (i.e., not just a randomized part of the training set), and comparing ground-truth outcomes with predictions.

A predictor can be a numerical variable, a categorical variable, a media variable (sound recordings, images, videos, etc.), or a text-based variable. Numerical and categorical variables correspond to structured data, whereas media data and text-based variables correspond to unstructured data. Media and text-based variables can be included in the ML process; however, these variables would require the use of specialized processing methods.

According to the state of the art^2–4^, the probability of missing data depends on the observed part of the dataset (*Y*_*obs*_), the missing part of the dataset (*Y*_*miss*_), the missing data mechanism (described by the parameter *ψ*), and the fact that the data are observed (*R* = 1) or not (*R* = 0):

1 $${{{\mathrm{Pr}}}}(R = 0|Y_{obs},Y_{miss},\psi )$$

Using the parameter *θ* to represent the dataset model, the probability of observed data can be represented by a density function:

2 $$f(Y_{obs},R|\theta ,\psi )$$

Missing data can be classified into three categories: missing completely at random (MCAR), missing at random (MAR), and missing not at random (MNAR)^4^. In the case of MCAR data, the probability of missing data is independent of the observed or missing data and depends solely on the missing data mechanism. Hence, the probability of missing data can be simplified, as follows:

3 $$\Pr \left( {R = 0{{{\mathrm{|}}}}Y_{obs},Y_{miss},\psi } \right) = {{{\mathrm{Pr}}}}(R = 0|\psi )$$

In the case of MAR data, the probability of missing data depends on observed data and the missing data mechanism. The probability of missing data can be then simplified, as follows:

4 $$\Pr \left( {R = 0{{{\mathrm{|}}}}Y_{obs},Y_{miss},\psi } \right) = {{{\mathrm{Pr}}}}(R = 0|Y_{obs},\psi )$$

Lastly, in the case of MNAR data, the probability of missing data depends on all the data and the missing data mechanism and so cannot be simplified.

All three types of missing data can be found in EM-related datasets. The MCAR data might be collected but not reported; for example, the triage nurse might forget to report the collected data. The MAR data might be not collected because they are not necessary or ethical with regard to the reason for attending the emergency department (ED); for instance, a capillary blood glucose measurement is not relevant for a young patient with an unremarkable medical history and who presents at the ED with a traumatic ankle injury. Lastly, MNAR data might be not collected because the collection is time-consuming (e.g., measurement of the respiratory rate).

Patients attend the ED for many different reasons. Firstly, several variables are systematically collected: administrative data, demographic data, the reason for attending, etc. Secondly, some variables are not collected systematically: blood pressure, pain scale, capillary blood glycemia, etc. In both cases, these variables can be categorical (e.g., the pain scale is from 0 to 10 and can include the additional category “not completed”) or numerical (e.g., the capillary blood glycemia value).

One can consider the simple situations summarized in Table 1; the reasons for missing data are all different and depend on several variables other than the capillary blood glycemia measurement. Many rules determine whether or not glycemia is measured. In line with this analysis, one can say that because the glycemia measurement depends on other variables, it could be missing at random. Given that the absence of these data is governed by medical and ethical rules, we further consider that the missing data are “data not purposely collected” (DNPC, a subtype of MAR data).

**Table 1:** Examples of how missing data can arise among patients attending the ED.

	Reason for coming	Medical history	Glycemia	Missing data	Reason
1	Minor mechanical trauma to the ankle	No	Not measured	Missing for a clinical decision: no Missing for a statistical analysis: yes	The reason for attending does not require a measurement
2	Minor mechanical trauma to the ankle	Type 1 diabetes	Measured	Missing for a clinical decision: no Missing for a statistical analysis: no	A diabetic patient’s glycemia must be measured, whatever the reason for attending
3	Minor ankle trauma due to fainting	No	Measured	Missing for a clinical decision: no Missing for a statistical analysis: no	The glycemia of a patient attending for fainting must be measured, whatever the medical history
4	Minor ankle trauma due to fainting	No	Measured Not reported	Missing for a clinical decision: no Missing for a statistical analysis: yes	The glycemia has been measured and clinically evaluated but is not reported in the software
5	Minor ankle trauma due to fainting	No	Not measured	Missing for a clinical decision: yes Missing for a statistical analysis: yes	The glycemia should have been measured but has not been. This is an error and constitutes real missing data

**Fig. 1:**
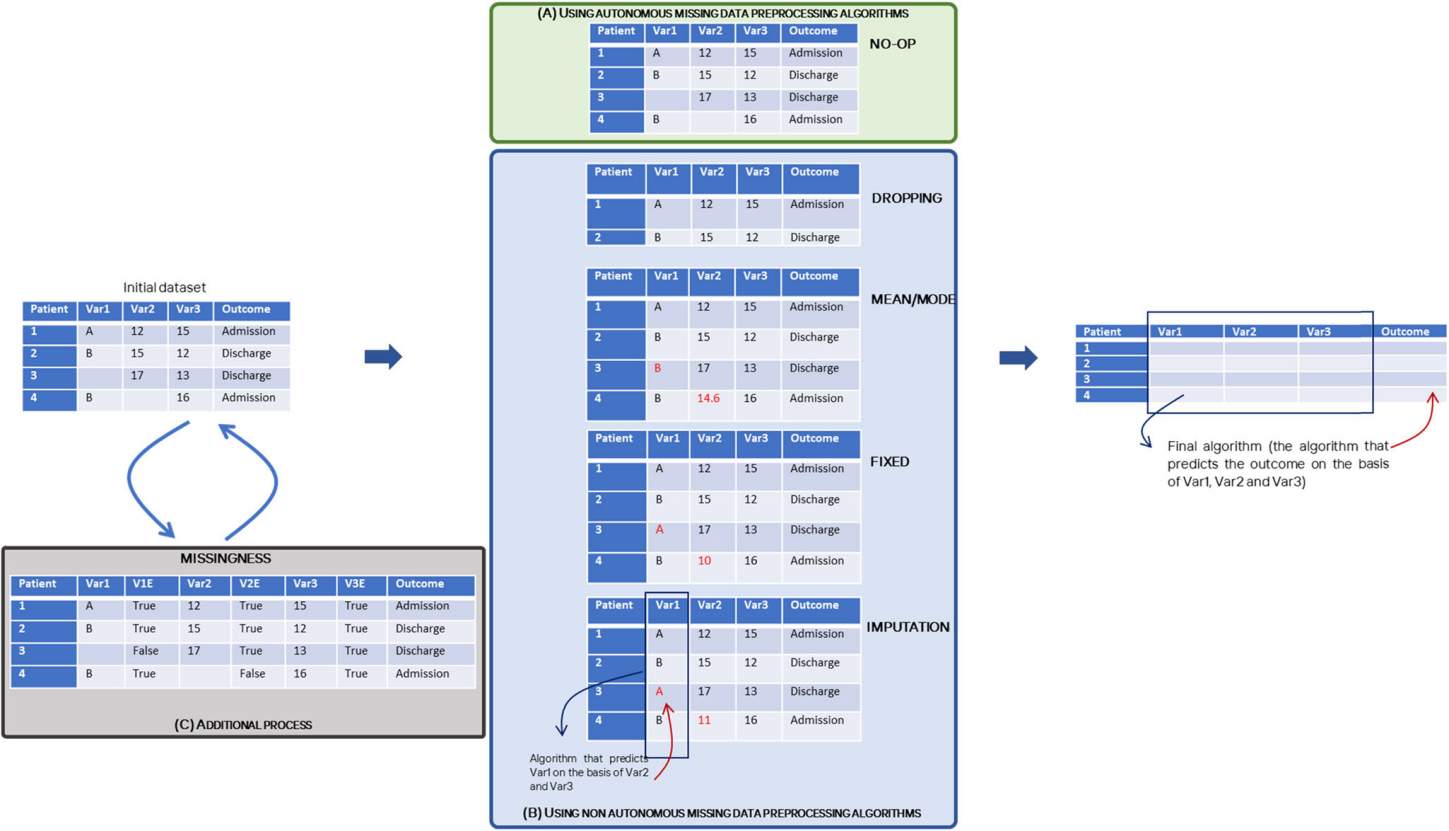
Missing data strategies applied before the final (predictive) algorithm. **A** Missing data strategy using autonomous missing data preprocessing algorithms. **B** Missing data strategies using non autonomous preprocessing algorithm. **C** Missing data strategies using additional process.

There are several algorithmic approaches; each has particular requirements with regard to the completeness of data. By design, basic logistic regression and deep-learning methods do not accept missing values as inputs in the low-level algorithm. Decision trees and derived methods (boosted decision trees, random forest, etc.) do not require a complete dataset, and missing data are interpreted as a specific category^5^. More complex logistic regression algorithms (such as the stochastic approximation version of expectation-maximization (SAEM) algorithm) also accept missing data with low bias and low standard error. This method consists in estimating the parameter *θ* in a logistic regression by maximizing the stochastic approximation of the simulated samples from the target in three steps: simulating an initial guess *θ*_0_, performing *k* loops of a stochastic approximation, and maximizing the estimation of *θ*_*k*_^6^. Filling in missing data is a common strategy when building a predictive model. In the field of healthcare, several strategies for dealing with missing numerical and categorical data have been applied: (A) using autonomous missing data preprocessing algorithms (i.e., in decision tree algorithms or SAEM, missing data are natively managed); the researcher does not do anything to the initial dataset and delegates the missing data management to the final ML algorithm (referred to as “no-op” hereafter), (B) not using autonomous preprocessing algorithms (basic logistic regression, and deep learning), which includes (i) a dropping strategy (removing patients with missing data) (ii) a mean imputation strategy (filling missing data with the variable’s mean value (a numerical variable) or mode value (a categorical value)), (iii) a fixed strategy (filling missing data with a fixed (physiological or reference) value), and (iv) an imputation strategy (imputing missing data with other ML techniques, such as logistic regression and chained equations, to impute missing data), and (C) additional process (missingness, i.e., adding a variable in the dataset which indicates whether the value is present or not). These strategies are shown in Fig. 1. Naemi et al. tested the impact of the data strategy by testing the same training process on the same initial dataset filled by different strategies: the AUROC of an extreme gradient-boosting decision tree model increased from 84% to 94% after completion of the dataset using Gaussian processing and synthetic minority oversampling techniques of class samples that were close to the borderline^7^. A complementary method consists in marking patients with missing data in novel variables in parallel with the application of another obligatory strategy. This additional information can improve the performance of ML methods.

The imputation strategy must take into account the ignorability of the missing data mechanism. Missing data are considered^8^ to be ignorable if they are MAR and if *ψ* and *θ* are distinct:

5 $$p\left( {\theta ,\psi } \right) = p(\theta )p(\psi )$$

If the missing data mechanism is ignorable, the distribution of *Y* is the same in the observed and non-observed groups^3^:

6 $$p\left( {Y{{{\mathrm{|}}}}Y_{obs},R = 1} \right) = p(Y|Y_{obs},R = 0)$$

In this case, there is no need to consider the missing data mechanism in the imputation, and a simple imputation model can complete most of the data without introducing bias. In contrast, the missing data mechanism must be included in a more complex imputation model (like the multiple imputation chained equation (MICE)) when the missing data mechanism is linked to the data themselves.

Given that reporting on an ML-based study is complex and highly technical, a group of methodologists developed the Transparent Reporting of a Multivariable Prediction Model for Individual Prognosis or Diagnosis (TRIPOD) statement. The TRIPOD statement is a checklist of 22 items that we consider to be essential for good reporting of studies developing or validating multivariable prediction models. It is intended to improve the transparency of reporting of a prediction model study, regardless of the predictive methods used^9^.

Wards and EDs differ in several respects. Firstly, the types of patients and reasons for admission are less specific in the ED than in wards: patients attend the ED for problems related to any specialty, whereas the patients admitted to ward often have a problem related to the ward’s specialty. Thus, not all data are systematically collected for all patients; it mostly depends on the patient’s reason for attendance. Secondly, the flow of patients attending the ED is continuous, whereas the flow in wards stops when all the beds are occupied. In the latter case, most of the patients stay for longer than one day. Thus, the time devoted to the patient differs, which impacts the data collection. When a patient attends the ED, some variables are always collected (e.g.: demographic data, admission and discharge dates, the final diagnosis, etc.) and others are only collected if dictated by the context or when possible (for instance, nurses might have to collect only essential data if the ED is overcrowded).

The distinctive context of EM yields a heterogeneous dataset. Missing data (also referred to as “DNPC”) do not constitute a bias per se because their absence is deliberate. Hence, the completion of DNPC is not the same as the completion of missing data in a standard, prospective study in which data should have been collected but were not. Thus, we posed the following question: how are DPNC addressed in ML-based studies within the field of EM?

The objectives of the present study were as follows. Firstly we sought to systematically review published DNPC strategies for building predictive models in the field of EM. Secondly, we sought to determine whether one strategy is clearly better than the others within this specific context.

## Results

### Study characteristics

**Fig. 2:**
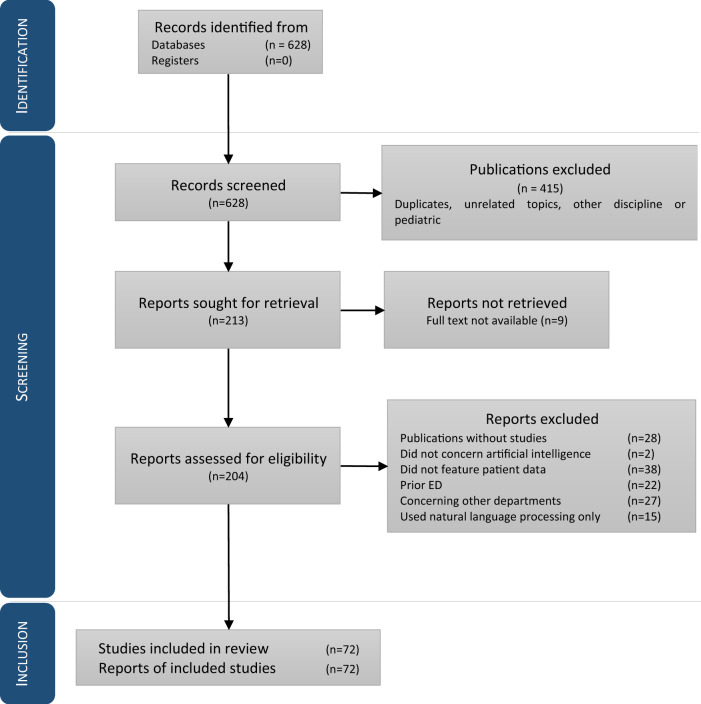
Identification, screening and inclusion of studies via databases and registers.

The literature search yielded 628 publications. After screening the titles, we selected 213 publications for analysis of the abstracts. A total of 141 publications were excluded on the basis of the abstract (*n* = 102) or the full text (*n* = 41). Hence, 72 full-text publications were included and analyzed (Fig. 2 and Supplementary Table 1). The mean (standard deviation) numbers of patients were 18,222,672 (1,119,747) overall, 5,635,600 (307,467) for the training sets, and 699,702 (60,254) for the validation sets. Only four publications complied with the TRIPOD checklist. None of the publications included a low risk of bias.

As outcome predicted by the models: a diagnosis is used in 25 publications (35%), mortality in 21 (29%), and the probability of admission in 21 (29%). The 12 remaining publications (16%) predicted a triage score or treatment administration. Eight publications (11%) predicted more than one outcome (Supplementary Table 2).

### Models and imputation strategies

Considering the type of model used to provide the best validation AUROC, the proportions of studies using XGBoost and logistic regression techniques grew by, respectively, 11% and 18% between 2018 and 2021. The use of deep learning models grew by 39% from 2018 to 2019 and then decreased by 52% to 2021 (Supplementary Fig. 1). In 2021, 13 (44.8%) of the 29 studies used the XGBoost as their best model and 10 (34.5%) used a logistic regression model. Thirty-seven studies (51.4%) tested multiple algorithms, and 35 (48.6%) only tested one. Of the 26 studies that tested the XGBoost, 16 (61.5%) tested it alone and 10 (38.5%) compared it with other algorithms. It is interesting to note that of the 16 studies that tested XGBoost alone, 9 (34.6%) used a missing data preprocessing strategy: one (11.1%) replaced missing data with fixed values, three (33.3%) dropped patients with missing data, three (33.3%) imputed with the mean, two (22.2%) using missingness, and two (22.2%) performed multiple imputation before training the XGBoost model.

**Fig. 3:**
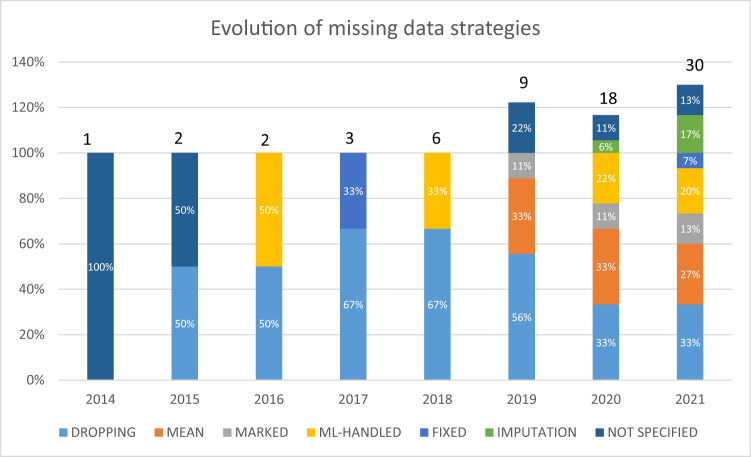
Change over time in the frequency of use of missing data strategies. NB: some publications used more than one strategy, and so the total can exceed 100%.

The missing data strategies applied in the publications are listed in Table 2. Forty-eight publications (67%) used a single strategy (the cells with bold values in Table 2), 14 publications (19%) used more than one strategy (the other cells in Table 2), and 10 publications (14%) did not specify the strategy. The most frequently applied strategy was dropping (30 publications; 41%), followed by the mean value method (17 publications; 23%), ML strategies (13 publications; 18%), imputation (6 publications; 8%), and the physiological/reference value method (3 publications; 4%). Four publications (5%) stated whether or not each individual value was missing in the study database; this approach is henceforth referred to as “Marked”. Ten publications (14%) did not specify the missing data strategy at all. The use of these strategies has clearly changed over time: the proportion of studies that used a dropping method fell from 67% in 2017 to 33% in 2021, whereas the proportion that used imputation rose from 6% in 2020 to 17% in 2021 (Fig. 3; some publications used more than one strategy, and so the total can exceed 100%).

**Table 2:** Missing data strategies applied in the reviewed publications.

Strategy	Total number of publications	Dropping	Mean	Missingness	No-op	Fixed	Imputation	Not defined	Mean AUROC
Dropping	30	**23**	2	2	1	1	1		0.87
Mean	17		**11**	2	1		1		0.84
Missingness	7			**0**	2		1		0.85
No-op	13				**9**				0.86
Fixed	3					**2**			0.89
Imputation	6						**3**		0.88
Not defined	10							**10**	0.84

The bold values correspond to the number of publications applying a single strategy. The other cells correspond to combinations of two missing data strategies. The mean AUROC values were computed only from publications that used a single strategy.

The most frequently reported patient-related predictors were age (70 publications; 97%) and gender (66 publications; 92%), although only 15 of the two groups of publications (21% and 23%, respectively) described their missing data. The missing data for other predictors are described in Table 3.

**Table 3:** Description of missing data in publications.

Predictor	Number of publications^a^	Total patients^b^	Missing description in publications^c^	%^d^	Total missing values^e^	%^f^
Age	70	24,554,988	15	21	13,109	0.05
Gender	66	24,297,072	15	23	1614	0.01
Race	27	9,401,259	6	22	30,691	0.33
Attendance mode	29	19,180,914	5	17	15,509	0.08
Reason for attendance	30	22,097,032	4	13	10,319	0.05
Time of visit	12	16,046,251	0	0		0.00
Day of visit	13	14,327,915	0	0		0.00
Triage	29	17,241,664	3	10	9166	0.05
GCS score	35	16,776,293	5	14	346,993	2.07
HR	56	18,959,043	11	20	82,330	0.43
RR	56	18,959,885	11	20	116,461	0.61
SBP	56	18,960,674	9	16	110,033	0.58
DBP	47	16,887,450	7	15	19,404	0.11
Blood oxygen saturation	45	7,678,163	7	16	749,390	9.76
Temperature	49	18,345,680	9	18	336,436	1.83
Pain scale	13	1,132,598	2	15	16,518	1.46
Body weight	10	238,607	3	30	6424	2.69
Prior ED visits	16	8,226,874	1	6	9250	0.11
Prior ICD-10 code	38	6,369,720	4	11	382	0.01
Hematology tests	24	1,595,185	6	25	1352	0.08
Blood electrolyte assay	25	1,837,740	3	12	956	0.05
Text data	4	469,776	2	50	6777	1.44
ECG	9	722,539	1	11	–	0.00

*GCS* Glasgow Coma Scale, *HR* heart rate, *RR* respiratory rate, *SBP* systolic blood pressure, *DBP* diastolic blood pressure, *ED* emergency department, *ICD-10* International Classification of Diseases, 10th Revision.

^a^The number of publications in which the predictor was used.

^b^The total number of patients included in the publications that used the predictor.

^c,d^The number or proportion of publications in which the missing data were specified.

^e,f^The number or proportion of patients for whom data on at least one predictor were missing.

## Discussion

The objective of the present review was to study the completion strategies used to address the DNPC problem in ML studies related to EM. The models described in the 72 included publications predicted a variety of outcomes and varied with regard to the predictor and the type and size of the patient population. These heterogeneous studies could not be compared directly and so any inferences must be applied with caution. However, most of the publications used the AUROC as the main performance indicator. Consequently, we used the AUROC as a comparator of our study, although no firm conclusions can be made on that basis.

Failure to describe missing data (rather than having missing data per se) can be considered as a significant source of bias. Since 2014, several publications have described how missing data should be reported^10^. For all predictors (other than text data), fewer than 25% of the reviewed publications described their missing data. The number of missing values was probably underestimated because of this under-description, which would have introduced significant bias into the interpretation. This proportion is nevertheless higher than that reported by a 2019 review of clinical pharmacy research^11^, in which only 19.7% of the selected publications mentioned how they handled missing data. We recommend explicitly specifying the amount of missing data and describing how missing data are handled. This information could be summarized in an explicit, methodological diagram (Supplementary Fig. 2). Independently of the strategy applied, an algorithm’s in fine performance is the most important result. Even when an imputation strategy poorly estimates the missing values, it might help to improve the in fine prediction. This corresponds to the objective of the present study.

The dropping strategy was the most frequently used strategy. This is probably because dropping is the easiest to implement: all patients with missing data are simply excluded. The dropping technique is the only one that does not require completion. Its main drawback is the decrease in the size of the dataset. However, this strategy could lead to hidden selection bias and might mean that the study population is no longer representative of the target population^4^. If patients are dropped because of MCAR data, then no bias would be introduced. Dropping patients because of MCAR is rare but must be documented; this is not often done, particularly in the field of medicine^12^. Some publications used a slightly more sophisticated threshold approach (e.g., only patients with more than a certain amount of missing data (e.g., 50%) are excluded) or composite strategies (Table 1). However, the associated AUROC values were high, which might indicate that the dropping strategy did not have a negative impact on the training stage. The other common strategy for handling missing data was the “no-op” strategy. Indeed, the decision tree family of algorithms deals perfectly with missing data by using different methods (for instance, the surrogate split, probabilistic split, block propagation, and missing incorporated in attribute (MIA))^13^. This strategy is easy to implement and does not require preprocessing but obliges the investigators to use an autonomous ML-based approach to missing data preprocessing.

In the particular context of DNPC, the use of the mean capillary blood glucose value might be influenced by selection bias, which might explain why the “mean value” strategy had the lowest AUROC, on average (Table 2). Other measurements of the same type include (for example) alcohol levels, the bladder volume, some biochemical assay (e.g., troponin and C-reactive protein). The selection bias associated with DNPC is accepted in the EM for ethical, organizational and economic reasons. The enforced measurement of glycemia for every patient (especially when it is as intrusive as a needle stick) is not ethical when it is medically irrelevant.

Imputation consists in training a predictive model to impute missing data from existing data in the same dataset, using a chaining equation algorithm. The most commonly used of these is the MICE^14^, which gave a slight advantage in the study by Faris et al.^15^. However, several other techniques are available, such as Gaussian processing and synthetic minority oversampling techniques. The mean AUROC computed from publications using this strategy appears to confirm Faris et al.’s observation. However, the imputation strategy might involve more complex preprocessing and thus requires more time and more computational resources—especially when the number of features is large. Various options in the MICE implementation package can speed up the imputation, albeit at the price of greater approximation. The fixed strategy consists in filling missing data by a constant value (often a physiological or reference value) for each predictor. This method is not expected to yield a higher mean AUROC than for the imputation method. The same value is considered for each patient, with no consideration of other variables. Hence, a diabetic with a missing capillary glucose value would be given a normal capillary glucose value, which is not intuitive. The “not defined” strategy referred to studies that did not describe how they managed missing data. However, the trained algorithm in these studies performed worst - indicating that not specifying the missing data strategy is not a quality indicator. The strategy used to manage missing data was related to the type of algorithm chosen. More specifically, if the researchers had chosen an autonomous missing data preprocessing algorithm, they do not have to manage the missing data themselves. However, we found that some of the researchers who used XGBoost still preferred to manage the missing data themselves (depending on which data were missing); this choice is domain-specific and requires the physician’s expertise.

The DNPC handling strategies have changed over time, as described in Fig. 3. We distinguished between three periods: a period lacking a specific strategy (before 2016), a period with a single strategy (from 2016 to 2018), and a period with combinations of strategies (from 2019 to present). During the first period, prior, the publications either did not describe the strategy applied or used the “dropping” strategy. During the second period, a single, basic strategy was applied: dropping, using a physiological constant for all patients, or a native algorithm. During the last period, investigators chose more complex methods and combined them: marking patients with DNPC, using the mean, or imputing DNPC from patients with no missing data. The proportion of publications using simple strategies (mean, ML, and dropping) decreased; conversely, the proportion using the most complex strategy (imputation) increased to 17% in 2021.

The data collected here do not enable us to affirm that one particular strategy is always clearly more effective than the others. However, with reference to the AUROC, the no-op strategy appears to produce the best results and is being increasingly used. SAEM^16^ is a more recently developed algorithm and was not frequently used in the included studies; it might, however, perform better than MICE imputation prior to a logistic regression^6^. Even though the fixed value and dropping strategies gave good results, their use became less frequent over time.

Perez-Lebel et al.’s benchmarking of the different strategies for dealing with missing data in real health databases demonstrated than (i) the MIA-boosted trees give the best predictive models at little cost (a no-op strategy), (ii) missingness is informative, and (iii) using MICE in a large database is very costly^17^. However, Austin et al.^18^ demonstrated that using MICE on clinical data is valuable—especially when the missing data mechanism is nonignorable.

In the publications reviewed here, the missing data strategies were applied to the whole dataset. We hypothesize that the best strategy is dependent on the variable. Based on this review and on our experience, we recommend keeping all patients (i.e., not applying a dropping strategy) if the study in question covers all patients attending the ED. Next, physicians with expertise in the included population could choose a specific strategy variable by variable. For example, some variables could be completed by applying decision rules based on medical expertise (e.g., the Glasgow coma scale should be rated as 15 out of 15 in many patients with minor injuries). If logistic regression can be performed, we recommend applying SAEM to the dataset containing the rest of the missing data. In other cases, we recommend using boosted trees that deal with missing data (such as MIA). If the missing data mechanism is nonignorable, the MICE algorithm should be seriously considered because it includes the missing data mechanism parameter in its model.

The publications did not always describe the missing data, and the quality of reporting varied from one study to another; these problems can lead to interpretation bias. We suggest formalizing the description of missing data in the “Material and methods” section of publications.

TRIPOD is the reference methodology for building a multivariable prediction model^9^. Its section 9 recommends describing the missing data. However, this recommendation has not been formalized. We therefore recommend the inclusion of a formal table that lists all the missing data and describes the strategy applied to each variable (Supplementary Fig. 2).

In studies with imbalanced outcomes (e.g., hospital admission for 70% of the patients and discharge for 30%), the AUROC might not be the best index of an algorithm’s performance. In such a case, it might be valuable to compute the area under the precision–recall curve. However, researchers should always be required to report the AUROC, since it is the most common index of performance.

The presentation of the strategies varied from one publication to another. Even though all publications reported on the development or use of a predictive model involving supervised ML, the flowcharts were not standardized. Furthermore, the missing data were not always described, and the train-test split was not always mentioned clearly. Accordingly, we propose an add-on to the TRIPOD checklist, based on Martinez et al.’s diagram^19^ (Supplementary Fig. 2). Having this type of diagram in an ML publication (even as an appendix) might help the reader.

The present study had several limitations. First, we only searched the PubMed databases; databases such as Google Scholar or IEEE Xplore were not used. However, PubMed indexes all the relevant journals in the field of EM, and the number and variety of publications indexed in PubMed were sufficient for a methodological review. The same query yielded more than 200,000 hits in Google Scholar and more than 1000 in IEEE Xplore. Second, we searched for publications in English only and so might have missed relevant publications in other languages. Third, we did not consider publications before 2000. However, research on missing data management did not develop until the 2000s, and the most significant developments occurred over the last two decades. Fourth, we conducted a systematic review of methods used in EM studies, rather than results or findings. We focused on missing data management in heterogeneous studies that could not be directly compared as evidenced by the risk of bias analysis. This study does not review all literature on missing data, nonignorable missing data or all existing methods to learn the missingness data mechanism. Fifth, we focused on the EM field only because of the specificities of the ED in comparison with other wards. Specifically, we considered the number of patients managed, the variable daily flow rate, the very short length of stay in the ED, and the great variety of reasons for attending. However, similar missing data mechanisms could be observed in other specialties. Lastly, we used the AUROC as a comparator, even though other indicators^20^ could be considered (e.g., accuracy, recall, specificity, precision, F-measure, the Matthews correlation coefficient, the mean absolute error, and the Brier score).

DNPC constitute the “missing data” in ML studies in the field of EM. Although DNPC have been handled more rigorously since 2020, the strategies used are still not exhaustive, systematic or homogeneous. The imputation strategy appears to yield the best results, but requires more time and computational resources; however, given the heterogeneity of the studies reviewed, we cannot say whether one missing data strategy was clearly better than another. To increase the quality and comparability of these studies, we recommend filling out the TRIPOD checklist for each new publication, summarizing the ML process in an explicit methodological diagram, and always reporting the AUROC (even when it is not the primary outcome).

## Method

### Design

We extracted data and reported our results in accordance with the Critical Appraisal and Data Extraction for Systematic Reviews of Prediction Modelling Studies (CHARMS)^21^ and the Preferred Reporting Items for Systematic Reviews and Meta-Analysis (PRISMA)^22^ guidelines (see the checklist in Supplementary Table 3). With regard to missing data strategies, we extracted the models, predictors, and outcomes from each publication. The protocol was not registered before the beginning of the study.

### Search strategy

In January 2022, we searched the PubMed database with the following Medical Subject Headings (MeSH): “(emergency medicine OR emergency service) AND (artificial intelligence OR machine learning)”.

### Inclusion and exclusion criteria

We assessed all publications in English concerning the construction or application of a predictive model for structured data in an adult ED. Duplicate studies and reviews were excluded, as were studies published before 2000. We excluded studies whose predictive model was based solely on natural language processing, given that the missing data are not typically handled in the same way as for structured data. Studies based solely on additional examinations (such as imaging or bacteriologic tests) and that lacked the clinical context were also excluded. Lastly, non-clinical studies (i.e., based solely on societal variables) and publications for which the full text was not available were also excluded.

### The study selection process

Two investigators (E.A. and D.A.G.) screened the PubMed search results on the basis of each publication title. Next, the two investigators read the abstracts and excluded ineligible studies. Lastly, the full-text versions of the remaining publications were read to determine whether the methodological details met the inclusion criteria.

### The data collection process

Data in the included publications (publication references, the relevant CHARMS items, missing data strategies, the dataset split, and best AUROC value for training and validation stages) were entered on an Excel® (Microsoft Corp., Cupertino, USA) spreadsheet by one investigator (E.A.) and reviewed by another (D.A.G.). If the publication did not specify how missing data were handled, we considered that the strategy was “not specified”. If the publication did not specify how the dataset had been split into a training set and a test set, we considered they used the typical 80:20 ratio. Lastly, we considered that the use of an independent dataset was obligatory for a validation step. When the dataset used to calculate an algorithm’s performance was not independent, we considered that the algorithm had been tested but not validated.

### The data analysis

We counted the missing data strategies applied in the reviewed publication in a first matrix table. Since multiple strategies could be applied in a publication, a publication can be counted twice. We considered the mean of the AUROC to describe the performance of the publication when a unique strategy was applied. We described the missing data in a second table by counting for each variable the number of publications, patients, and missing data. We reported the publication using the Prediction model Risk Of Bias ASsessment Tool (PROBAST)^23^.

## Supplementary information

Supplementary information
