# Supplementary material for: Predictive models in emergency medicine and their missing data strategies: a systematic review
Source: NPJ Digit Med. 2023 Feb 23;6:28. doi: 10.1038/s41746-023-00770-6 (PMC9950346; doi:10.1038/s41746-023-00770-6)
Supplement: Supplementary file 1 — Supplementary information [file 41746_2023_770_MOESM1_ESM.pdf]

Supplementary Information

Supplementary Table 1. Details of reviewed publications. Coronavirus disease 2019 (COVID), intensive care unit (ICU), neurological intensive care unit (NICU), electrocardiogram (ECG), major adverse cardiac event (MACE), International Classification of Disease, 10th Edition (ICD-10), computed tomography (CT), ST-elevation myocardial infarction (STEMI), non ST-elevation myocardial infarction (NSTEMI), quick Sepsis-related Organ Failure Assessment (qSOFA).

Title	Reference	Best model	Main predictors	Missing data strategy	Outcome
A 12-hospital prospective evaluation of a clinical decision support prognostic algorithm based on logistic regression as a form of machine learning to facilitate decision making for patients with suspected COVID-19	Lupei et al., 2022[24]	Logistic regression	Demographic variables, prior ICD-10 codes, prior medication, and vital signs	DROPPING	Severe COVID infection (ICU admission) or NIV or mortality
Using Machine Learning to Make Predictions in Patients Who Fall	Young et al., 2021[25]	Logistic regression	Demographic variables, prior ICD-10 codes, triage score, and vital signs	MEAN	Mortality and discharge at home
Supervised classification techniques for prediction of mortality in adult patients with sepsis	Rodriguez et al., 2021[26]	SVM	Demographic variables, triage score, vital signs, laboratory data, and treatments	DROPPING	In-hospital mortality
Predictors of emergency department opioid administration and prescribing: A machine learning approach	McCann-Pineo et al., 2021[27]	Logistic regression	Demographic variables, socio-economic variables, prior ICD-10 codes, complaints, vital signs, triage score, and treatments	NOT DEFINED	Prescription of opioid
Machine learning-based models to support decision-making in emergency department triage for patients with suspected cardiovascular disease	Jiang et al., 2021[28]	XGBoost	Demographic variables, vital signs and triage score	NO-OP	Nurse triage level
Machine learning models predict coagulopathy in spontaneous intracerebral hemorrhage patients in ER	Zhu et al., 2021[29]	Random forest	Demographic variables, prior ICD-10 codes, triage score, vital signs, laboratory data, imaging and treatments	DROPPING	Coagulopathy
Improving ED Emergency Severity Index Acuity Assignment Using Machine Learning and Clinical Natural Language Processing	Ivanov et al., 2021[30]	XGBoost	Demographic variables, socio-economic variables, prior ICD-10 codes, vital signs, and text data	DROPPING and MISSINGNESS	Triage nurse

Title	Reference	Best model	Main predictors	Missing data strategy	Outcome
Predicting adult neuroscience intensive care unit admission from emergency department triage using a retrospective, tabular-free text machine learning approach	Klang et al., 2021[31]	XGBoost	Demographic variables, prior ICD-10 codes, prior attendance, vital signs, and text data	DROPPING missing triage notes and NO-OP	NICU admission
A comparison of machine learning models versus clinical evaluation for mortality prediction in patients with sepsis	Van Doorn et al., 2021[32]	XGBoost	Demographic variables, prior ICD-10 codes, vital signs, and laboratory data	NO-OP and MISSINGNESS	Death at 31d
A Machine Learning Prediction Model of Respiratory Failure Within 48 Hours of Patient Admission for COVID-19: Model Development and Validation	Bolourani et al., 2021[33]	XGBoost	Demography, prior ICD-10 codes, weight, vital signs, and laboratory data	IMPUTATION for numerical values and added MISSINGNESS category missing for categorical values	Intubation and mechanical ventilation within 48h
Rapid triage for COVID-19 using routine clinical data for patients attending hospital: development and prospective validation of an artificial intelligence screening test	Soltan et al., 2021[34]	XGBoost	Demographic variables, prior ICD-10 codes, vital signs, and laboratory data	Multiple IMPUTATION strategies, population MEAN, population median and age-based imputation, were used to impute missing data	COVID-19
Early risk assessment for COVID-19 patients from emergency department data using machine learning	Heldt et al., 2021[35]	Logistic regression	Demographic variables, vital signs, laboratory, and text data	MEAN	AICU admission, ventilation, mortality
Assessment of Thoracic Pain Using Machine Learning: A Case Study from Baja California, Mexico	Rojas-Mendiza, 2021[36]	Logistic regression	Demographic variables, prior ICD-10 codes, vital signs, and ECG	NOT DEFINED	Cardiac thoracic pain
Machine learning methods to predict mechanical ventilation and mortality in patients with COVID-19	Yu et al., 2021[37]	XGBoost	Demographic variables, prior ICD-10 codes, vital signs, laboratory data, and treatments	Not asked OR NO-OP or MEAN for numerical	Mortality and mechanical ventilation
Enhancement in Performance of Septic Shock Prediction Using National Early Warning Score, Initial Triage Information, and Machine Learning Analysis	Yun et al., 2021[38]	XGBoost	Demographic variables, vital signs, laboratory data, and treatments	DROPPING	Septic shock within 24 h
Clinical Features of Emergency Department Patients from Early COVID-19 Pandemic that Predict SARS-CoV-2 Infection: Machine-learning Approach	Chou et al., 2021[39]	Random forest	Demographic variables, prior ICD-10 codes, weight, and vital signs	DROPPING	COVID+

Title	Reference	Best model	Main predictors	Missing data strategy	Outcome
Utilizing machine learning dimensionality reduction for risk stratification of chest pain patients in the emergency department	Liu et al., 2021[40]	Logistic regression	Demographic variables, prior ICD-10 codes, vital signs, laboratory data, ECG and treatments	NOT DEFINED	Major adverse cardiac event
Predicting outcomes in older ED patients with influenza in real time using a big data-driven and machine learning approach to the hospital information system	Tan et al., 2021[41]	Logistic regression	Demographic variables, prior ICD-10 codes, vital signs and laboratory data	FIXED: normal values for laboratory variables and physiological values for vital signs and DROPPING for others	Admission or complications, or ICU or mortality
Clinical factors associated with rapid treatment of sepsis	Song et al., 2021[42]	XGBoost	Demographic variables, prior ICD-10 codes, vital signs, clinical data, laboratory data	FIXED: 0 for categorical and NA for numerical	Hypotensive or nonhypotensive triggered patient
Development and Validation of Machine Learning Models to Predict Admission From Emergency Department to Inpatient and Intensive Care Units	Fenn et al., 2021[43]	XGBoost	Demographic variables, prior ICD-10 codes, prior attendance, vital signs, triage score, laboratory data, and treatments	MEAN and MISSINGNESS	Hospital admission, ICU or discharge
Machine learning for developing a prediction model of hospital admission of emergency department patients: Hype or hope?	De Hond et al., 2021[44]	XGBoost	Demographic variables, prior ICD-10 codes, prior attendance, complaints, triage score, vital signs, and laboratory data	DROPPING for some variables then specific MICE IMPUTATION for continuous variables (and MISSINGNESS) and specific categories for categorical values	Hospital admission
Developing a stroke alert trigger for clinical decision support at emergency triage using machine learning	Sung et al., 2021[45]	Logistic regression	Demographic variables, prior ICD-10 codes, complaints, triage score, vital signs, and ECG	MEAN	Stroke
Predicting mortality among septic patients presenting to the emergency department-a cross sectional analysis using machine learning	Karlsson et al., 2021[46]	Random forest	Demographic variables, prior ICD-10 codes, vital signs, clinical data, and text data	DROPPING	Mortality
Developing machine learning models to personalize care levels among emergency room patients for hospital admission	Nguyen et al., 2021[47]	XGBoost	Demographic variables, prior ICD-10 codes, vital signs, and laboratory data	MICE IMPUTATION	ICU and hospital admission
Large Vessel Occlusion Prediction in the Emergency Department with National Institutes of Health Stroke Scale Components: A Machine Learning Approach	Huo et al., 2021[48]	Gaussian Process	Prior ICD-10 codes, and clinical data	NOT DEFINED	Large vessel occlusion

Title	Reference	Best model	Main predictors	Missing data strategy	Outcome
Development and Assessment of an Interpretable Machine Learning Triage Tool for Estimating Mortality After Emergency Admissions	Xie et al., 2021[49]	Logistic regression	Demographic variables, prior ICD-10 codes, and vital signs	MEAN	Mortality
Machine learning based early mortality prediction in the emergency department	Li et al., 2021[50]	Logistic regression	Demographic variables, vital signs, and laboratory data	MEAN	Early mortality
Prediction of hospitalization using artificial intelligence for urgent patients in the emergency department	Lee et al., 2021[51]	Neural network	Demographic variables, prior ICD-10 codes, complaints, and vital signs	DROPPING	Hospital admission
A machine learning model to predict critical care outcomes in patient with chest pain visiting the emergency department	Wu et al., 2021[52]	Logistic regression	Demographic variables, prior ICD-10 codes, weight, vital signs, ECG, laboratory data and treatments	RANDOM FOREST IMPUTATION	Critical care
Machine learning for outcome predictions of patients with trauma during emergency department care	Cardosi et al., 2021[53]	XGBoost	Demographic variables, prior ICD-10 codes, complaints, and vital signs	NO-OP	Mortality
Early prediction of in-hospital death of COVID-19 patients: a machine-learning model based on age, blood analyses, and chest x-ray score	Garrafa et al., 2021[54]	Light gradient boosting machine	Demographic variables, laboratory data, and imaging	NO-OP	Mortality
A comparison of logistic regression models with alternative machine learning methods to predict the risk of in-hospital mortality in emergency medical admissions via external validation	Faisal et al., 2020[55]	Logistic regression	Demographic variables, clinical data and laboratory data	DROPPING	Mortality
Developing neural network models for early detection of cardiac arrest in emergency department	Jang et al., 2020[56]	Neural network	Demographic variables, complaints, and vital signs	NOT DEFINED	Cardiac arrest
Promoting head CT exams in the emergency department triage using a machine learning model	Klang et al., 2020[57]	XGBoost	Demographic variables, prior attendance, complaints, triage score, and vital signs	NO-OP	Use of CT

Title	Reference	Best model	Main predictors	Missing data strategy	Outcome
A Gradient Boosting Machine Learning Model for Predicting Early Mortality in the Emergency Department Triage: Devising a Nine-Point Triage Score	Klug et al., 2020[58]	XGBoost	Demographic variables, prior ICD-10 codes, prior attendance, complaints, triage score, and vital signs	NO-OP	Mortality
Machine learning for prediction of septic shock at initial triage in emergency department	Kim et al., 2020[59]	Light gradient boosting machine	Demographic variables, complaints, vital signs, and laboratory data	DROPPING if >50% missing values, then MEAN	Septic shock within 24 h
A new risk stratification score for patients with suspected cardiac chest pain in emergency departments, based on machine learning	Mao et al., 2020[60]	XGBoost	Not reported	NO-OP	MACE at 7 days
Predicting Intensive Care Unit admission among patients presenting to the emergency department using machine learning and natural language processing	Fernandes et al., 2020[61]	Logistic regression	Demographic variables, prior attendance, complaints, triage score, and vital signs	DROPPING	ICU admission
Risk of mortality and cardiopulmonary arrest in critical patients presenting to the emergency department using machine learning and natural language processing	Fernandes et al., 2020[62]	XGBoost	Demographic variables, prior attendance, triage score and vital signs	MEAN and MISSINGNESS	Cardiac arrest within 24 h
Emergency department disposition prediction using a deep neural network with integrated clinical narratives and structured data	Chen et al., 2020[63]	Neural network	Demographic variables, complaints, weight, vital signs and text data	DROPPING	Hospital admission
Predicting hospital admission for older emergency department patients: Insights from machine learning	Mowbray et al., 2020[64]	XGBoost	Demographic variables, prior attendance, complaints, triage score	KNN IMPUTATION	Hospital admission
Chest CT for triage during COVID-19 on the emergency department: myth or truth?	Hermans et al., 2020[65]	Logistic regression	Demographic variables, triage score, vital signs, laboratory data, imaging, and treatments	DROPPING	COVID+
Early Prediction of Acute Kidney Injury in the Emergency Department With Machine-Learning Methods Applied to Electronic Health Record Data	Martinez et al., 2020[22]	Random forest	Demographic variables, prior ICD-10 codes, complaints, vital signs, and laboratory data	NO-OP and MISSINGNESS	Acute kidney injury

Title	Reference	Best model	Main predictors	Missing data strategy	Outcome
A multicenter mixed-effects model for inference and prediction of 72-h return visits to the emergency department for adult patients with trauma-related diagnoses	Yaghmaei et al., 2020[66]	XGBoost	Demographic variables, prior attendance, usage of hospital, diagnosis, and treatments	NOT DEFINED	Reattendance within 72 h
Real-time AI prediction for major adverse cardiac events in emergency department patients with chest pain	Zhang et al., 2020[67]	Random forest	Demographic variables, prior ICD-10 codes, weight, and laboratory data	MEAN	Acute myocardial infarction and all-cause mortality
Emergency department routine data and the diagnosis of acute ischemic heart disease in patients with atypical chest pain	Kim et al., 2020[68]	XGBoost	Demographic variables, vital signs, and laboratory data	MEAN	Acute ischemic heart disease
Development and External Validation of a Machine Learning Tool to Rule Out COVID-19 Among Adults in the Emergency Department Using Routine Blood Tests: A Large, Multicenter, Real-World Study	Plante et al., 2020[69]	XGBoost	Demographic variables and laboratory data	DROPPING	COVID+
Personalized Predictive Models for Identifying Clinical Deterioration Using LSTM in Emergency Departments	Naemi et al., 2020[70]	Neural network	Demographic variables, and vital signs	MEAN	Triage nurse
Using machine learning tools to predict outcomes for emergency department intensive care unit patients	Zhai et al., 2020[71]	XGBoost	Demographic variables, vital signs, and laboratory data	MEAN	Mortality
The Sydney Triage to Admission Risk Tool (START2) using machine learning techniques to support disposition decision-making	Rendell et al., 2019[72]	Logistic regression	Demographic variables, prior attendance, complaints, triage score and vital signs	DROPPING	Hospital admission as an inpatient
Artificial intelligence to predict needs for urgent revascularization from 12-leads electrocardiography in emergency patients	Goto et al., 2019[73]	Neural network	ECG	NOT DEFINED	Revascularization
Heart rate variability based machine learning models for risk prediction of suspected sepsis patients in the emergency department	Chiew et al., 2019[74]	XGBoost	Demographic variables, vital signs, and ECG,	NOT DEFINED	qSOFA

Title	Reference	Best model	Main predictors	Missing data strategy	Outcome
Emergency department triage prediction of clinical outcomes using machine learning models	Raita et al., 2019[75]	Neural network	Demographic variables, prior ICD-10 codes, complaints, triage score and vital signs	DROPPING	Critical care
Advanced diagnostic imaging utilization during emergency department visits in the United States: A predictive modeling study for emergency department triage	Zhang et al., 2019[76]	Neural network	Demographic variables, socio-economic variables, prior ICD-10 codes, prior attendance, complaints, triage score, vital signs and text data	MEAN	Use of advanced diagnostic imaging
An artificial intelligence approach to early predict non-ST-elevation myocardial infarction patients with chest pain	Wu et al., 2019[77]	Neural network	Demographic variables, prior ICD-10 codes, vital signs, ECG and laboratory data	DROPPING if > 50% missing values then MEAN	Acute coronary syndrome (Unstable angina/NSTEMI/STEMI)
Training and Interpreting Machine Learning Algorithms to Evaluate Fall Risk After Emergency Department Visits	Patterson et al., 2019[78]	XGBoost	Demographic variables, prior attendance, prior medications, and laboratory data	DROPPING for numerical variables and MISSINGNESS for categorical variables	Reattendance within 6 months
Training machine learning models to predict 30-day mortality in patients discharged from the emergency department: a retrospective, population-based registry study	Blom et al., 2019[79]	Logistic regression	Demographic variables, triage score, physician experience	DROPPING	Mortality
Predicting emergency department orders with multilabel machine learning techniques and simulating effects on length of stay	Hunter-Zinck, 2019[80]	Neural network	Demographic variables, complaints, and vital signs	MEAN	
Machine-Learning-Based Electronic Triage More Accurately Differentiates Patients With Respect to Clinical Outcomes Compared With the Emergency Severity Index	Levin et al., 2018[81]	Random forest	Demographic variables, complaints, and vital signs	DROPPING	Triage
Derivation and validation of different machine-learning models in mortality prediction of trauma in motorcycle riders: a cross-sectional retrospective study in southern Taiwan	Kuo et al., 2018[82]	SVM	Demographic variables, prior ICD-10 codes, vital signs, and laboratory data	DROPPING	Mortality
Multicentre validation of a sepsis prediction algorithm using only vital sign data in the emergency department, general ward and ICU	Mao et al., 2018[83]	XGBoost	Demographic variables and vital signs	DROPPING	Sepsis

Title	Reference	Best model	Main predictors	Missing data strategy	Outcome
The Impact of Risk Standardization on Variation in CT Use and Emergency Physician Profiling	Taylor et al., 2018[84]	Logistic regression	Demographic variables, prior ICD-10 codes, complaints, triage score, vital signs, and laboratory data	NO-OP	Use of CT
Predicting hospital admission at emergency department triage using machine learning	Hong et al., 2018[85]	XGBoost	Demographic variables, prior ICD-10 codes, complaints, triage score, ECG, and laboratory data	NO-OP for the best model (XGBoost), IMPUTATION for other tested models	Hospital admission
Validation of deep-learning-based triage and acuity score using a large national dataset	Kwon et al., 2018[86]	Neural network	Demographic variables, complaints, vital signs and clinical data	DROPPING	Mortality
Artificial neural networks: Predicting head CT findings in elderly patients presenting with minor head injury after a fall	Dusenberry et al., 2017[87]	Neural network	Demographic variables, complaints, clinical data and treatments	DROPPING	Hemorrhage on a CT scan
Creating an automated trigger for sepsis clinical decision support at emergency department triage using machine learning	Horng et al., 2017[88]	SVM	Demographic variables, complaints, and vital sign	FIXED PHYSIOLOGICAL	Infection
Prediction of Emergency Department Hospital Admission Based on Natural Language Processing and Neural Networks	Zhang et al., 2017[89]	Logistic regression	Demographic variables, prior ICD-10 codes, prior attendance, complaints, triage score, and vital signs	DROPPING	Hospital admission
Prediction of In-hospital Mortality in Emergency Department Patients With Sepsis: A Local Big Data-Driven, Machine Learning Approach	Taylor et al., 2016[90]	Random forest	Demographic variables, prior ICD-10 codes, complaints, triage, vital signs, and laboratory data	NO-OP	Mortality
Building a Decision Support System for Inpatient Admission Prediction With the Manchester Triage System and Administrative Check-in Variables	Zlotnik et al., 2016[91]	Neural network	Demographic variables, prior attendance, complaints, and triage score	DROPPING	Hospital admission
Evaluation of a hospital admission prediction model adding coded chief complaint data using neural network methodology	Handly et al., 2015[92]	Neural network	Demographic variables, and triage score	NOT DEFINED	Hospital admission

Title	Reference	Best model	Main predictors	Missing data strategy	Outcome
Are Mortality and Acute Morbidity in Patients Presenting With Nonspecific Complaints Predictable Using Routine Variables?	Jenny et al., 2015[93]	Logistic regression	Demographic variables, prior ICD-10 codes, triage score, clinical data, laboratory data and treatments	DROPPING	Mortality
Prediction of adverse cardiac events in emergency department patients with chest pain using machine learning for variable selection	Liu et al., 2014[94]	Random forest	Demographic variables, vital signs, and ECG	NOT DEFINED	MACE at 7 days

Supplementary Table 2. Number of publications for each type of outcome (primary or secondary). Area under the receiver operator curve (AUROC).

Outcome	# of publications	MORTALITY	ADMISSION	TRIAGE	DIAGNOSIS	TREATMENT	Mean AUROC
MORTALITY	21	14	3		2	2	0.88
ADMISSION	21		17			1	0.83
TRIAGE	4			4			0.92
DIAGNOSIS	25				23		0.88
TREATMENT	8					5	0.85

Supplementary Figure 1. Types of model, by year

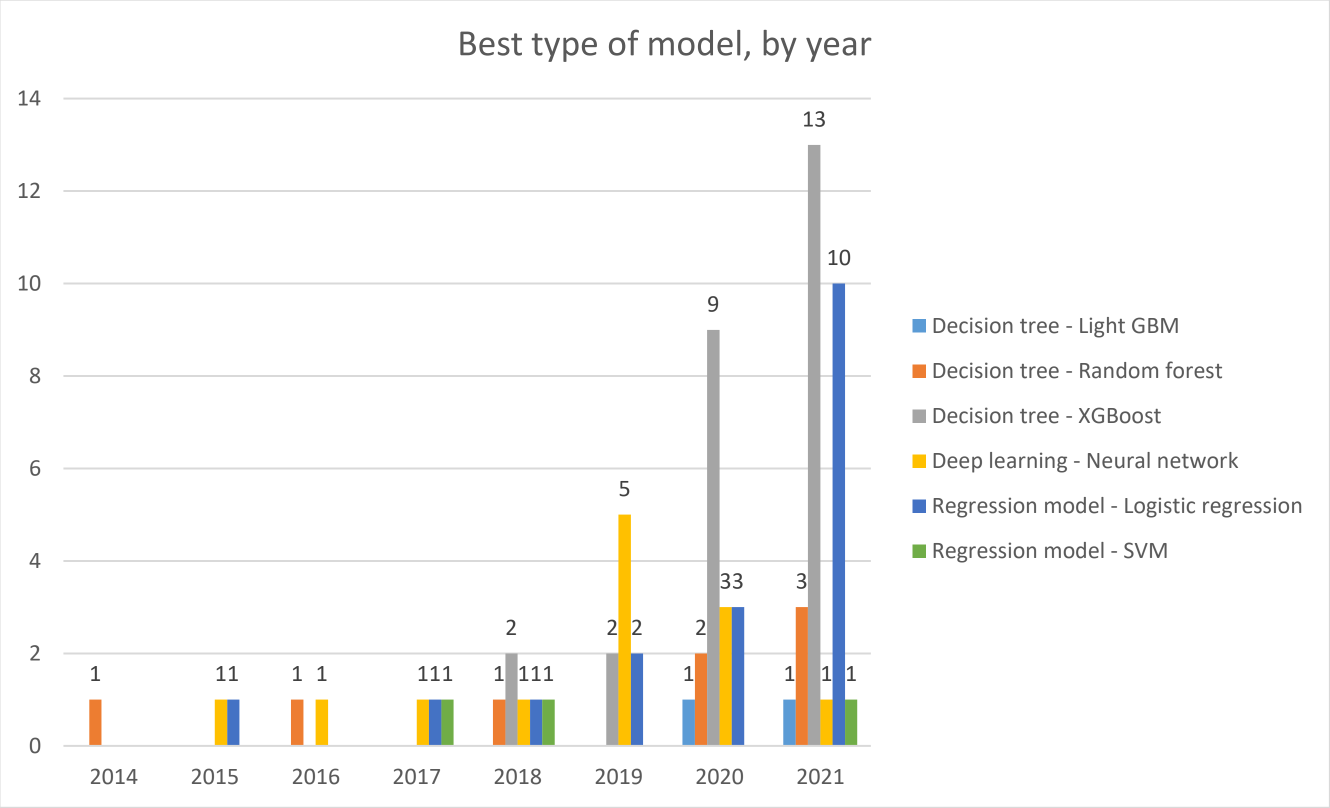

Supplementary Figure 2. An explicit diagram of the overall missing data strategy, adapted from Martinez et al.[22]

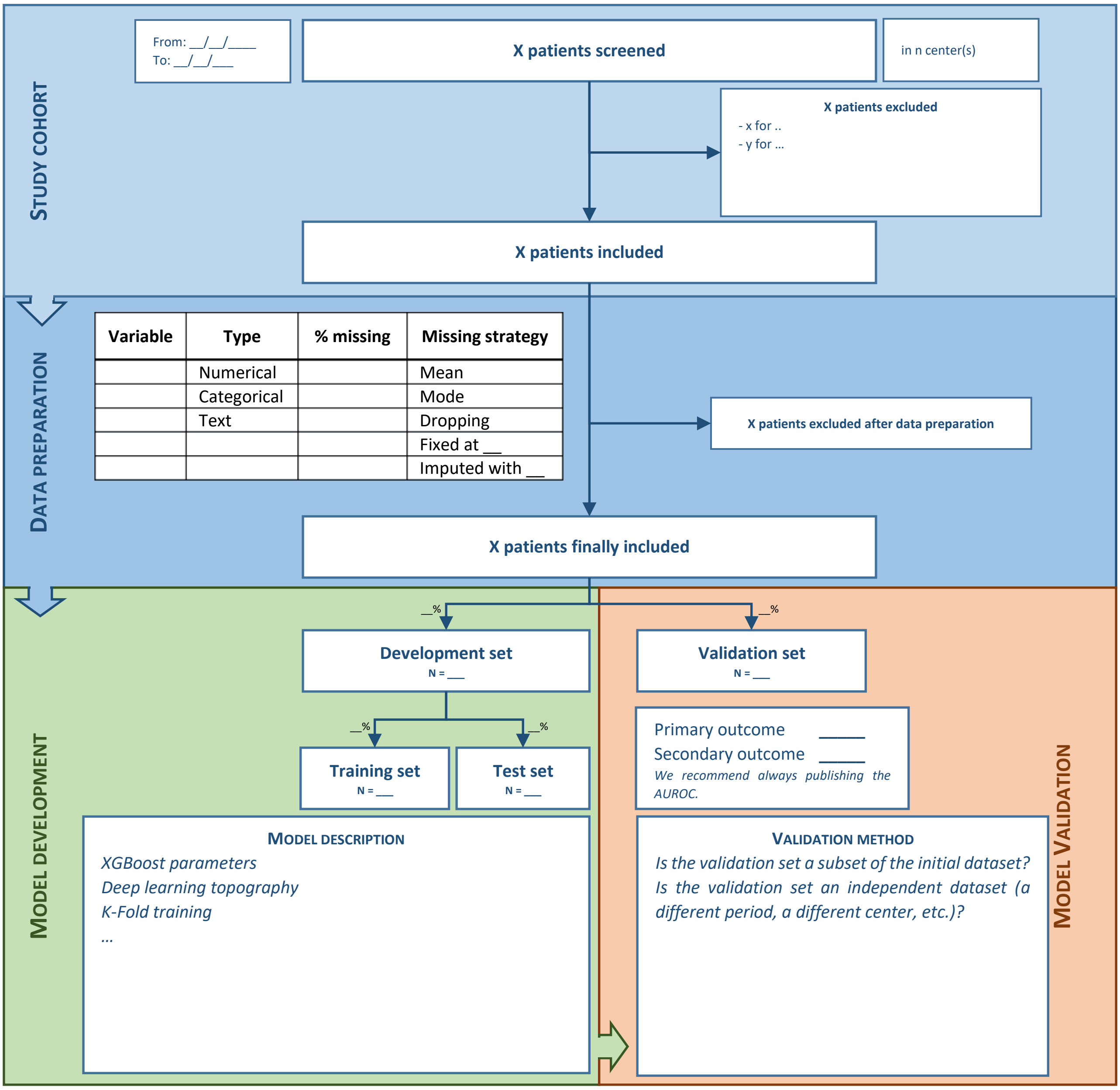

Supplementary Table 3. The PRISMA Checklist

Section and Topic	Item #	Checklist item	Location where item is reported
**TITLE**			
Title	1	Identify the report as a systematic review.	p. 1
**ABSTRACT**			
Abstract	2	See the PRISMA 2020 for Abstracts checklist.	Below this table
**INTRODUCTION**			
Rationale	3	Describe the rationale for the review in the context of existing knowledge.	p. 3
Objectives	4	Provide an explicit statement of the objective(s) or question(s) the review addresses.	p. 5
**METHODS**			
Eligibility criteria	5	Specify the inclusion and exclusion criteria for the review and how studies were grouped for the syntheses.	p. 10
Information sources	6	Specify all databases, registers, websites, organisations, reference lists and other sources searched or consulted to identify studies. Specify the date when each source was last searched or consulted.	p. 10
Search strategy	7	Present the full search strategies for all databases, registers and websites, including any filters and limits used.	p. 10
Selection process	8	Specify the methods used to decide whether a study met the inclusion criteria of the review, including how many reviewers screened each record and each report retrieved, whether they worked independently, and if applicable, details of automation tools used in the process.	p. 10
Data collection process	9	Specify the methods used to collect data from reports, including how many reviewers collected data from each report, whether they worked independently, any processes for obtaining or confirming data from study investigators, and if applicable, details of automation tools used in the process.	p. 10
Data items	10a	List and define all outcomes for which data were sought. Specify whether all results that were compatible with each outcome domain in each study were sought (e.g. for all measures, time points, analyses), and if not, the methods used to decide which results to collect.	p. 10
	10b	List and define all other variables for which data were sought (e.g. participant and intervention characteristics, funding sources). Describe any assumptions made about any missing or unclear information.	p. 10
Study risk of bias assessment	11	Specify the methods used to assess risk of bias in the included studies, including details of the tool(s) used, how many reviewers assessed each study and whether they worked independently, and if applicable, details of automation tools used in the process.	p. 9
Effect measures	12	Specify for each outcome the effect measure(s) (e.g. risk ratio, mean difference) used in the synthesis or presentation of results.	p. 10
Synthesis methods	13a	Describe the processes used to decide which studies were eligible for each synthesis (e.g. tabulating the study intervention characteristics and comparing against the planned groups for each synthesis (item #5)).	p. 10
	13b	Describe any methods required to prepare the data for presentation or synthesis, such as handling of missing summary statistics, or data conversions.	p. 10
	13c	Describe any methods used to tabulate or visually display	p. 10

Section and Topic	Item #	Checklist item	Location where item is reported
		results of individual studies and syntheses.	
	13d	Describe any methods used to synthesize results and provide a rationale for the choice(s). If meta-analysis was performed, describe the model(s), method(s) to identify the presence and extent of statistical heterogeneity, and software package(s) used.	p. 10
	13e	Describe any methods used to explore possible causes of heterogeneity among study results (e.g. subgroup analysis, meta-regression).	p. 10
	13f	Describe any sensitivity analyses conducted to assess robustness of the synthesized results.	p. 10
Reporting bias assessment	14	Describe any methods used to assess risk of bias due to missing results in a synthesis (arising from reporting biases).	p. 10
Certainty assessment	15	Describe any methods used to assess certainty (or confidence) in the body of evidence for an outcome.	p. 10
**RESULTS**			
Study selection	16a	Describe the results of the search and selection process, from the number of records identified in the search to the number of studies included in the review, ideally using a flow diagram.	Figure 2
	16b	Cite studies that might appear to meet the inclusion criteria, but which were excluded, and explain why they were excluded.	Figure 2
Study characteristics	17	Cite each included study and present its characteristics.	Supplementary table 1
Risk of bias in studies	18	Present assessments of risk of bias for each included study.	*p. 6*
Results of individual studies	19	For all outcomes, present, for each study: (a) summary statistics for each group (where appropriate) and (b) an effect estimate and its precision (e.g. confidence/credible interval), ideally using structured tables or plots.	Supplementary table 1
Results of syntheses	20a	For each synthesis, briefly summarise the characteristics and risk of bias among contributing studies.	p. 6
	20b	Present results of all statistical syntheses conducted. If meta-analysis was done, present for each the summary estimate and its precision (e.g. confidence/credible interval) and measures of statistical heterogeneity. If comparing groups, describe the direction of the effect.	Table 2<br>Table 3
	20c	Present results of all investigations of possible causes of heterogeneity among study results.	Table 2
	20d	Present results of all sensitivity analyses conducted to assess the robustness of the synthesized results.	Table 2
Reporting biases	21	Present assessments of risk of bias due to missing results (arising from reporting biases) for each synthesis assessed.	Table 3
Certainty of evidence	22	Present assessments of certainty (or confidence) in the body of evidence for each outcome assessed.	Supplementary table 1
**DISCUSSION**			
Discussion	23a	Provide a general interpretation of the results in the context of other evidence.	p. 6
	23b	Discuss any limitations of the evidence included in the review.	p. 6
	23c	Discuss any limitations of the review processes used.	p. 9
	23d	Discuss implications of the results for practice, policy, and future research.	p. 9
**OTHER INFORMATION**			

Section and Topic	Item #	Checklist item	Location where item is reported
Registration and protocol	24a	Provide registration information for the review, including register name and registration number, or state that the review was not registered.	p. 9
	24b	Indicate where the review protocol can be accessed, or state that a protocol was not prepared.	p. 9
	24c	Describe and explain any amendments to information provided at registration or in the protocol.	*p. 9*
Support	25	Describe sources of financial or non-financial support for the review, and the role of the funders or sponsors in the review.	p. 11
Competing interests	26	Declare any competing interests of review authors.	p. 11
Availability of data, code and other materials	27	Report which of the following are publicly available and where they can be found: template data collection forms; data extracted from included studies; data used for all analyses; analytic code; any other materials used in the review.	*Supplementary table 1*

Section and Topic	Item #	Checklist item	Reported (Yes/No)
**TITLE**			
Title	1	Identify the report as a systematic review.	Yes
**BACKGROUND**			
Objectives	2	Provide an explicit statement of the main objective(s) or question(s) the review addresses.	Yes
**METHODS**			
Eligibility criteria	3	Specify the inclusion and exclusion criteria for the review.	Yes
Information sources	4	Specify the information sources (e.g. databases, registers) used to identify studies and the date when each was last searched.	Yes
Risk of bias	5	Specify the methods used to assess risk of bias in the included studies.	Yes
Synthesis of results	6	Specify the methods used to present and synthesise results.	Yes
**RESULTS**			
Included studies	7	Give the total number of included studies and participants and summarise relevant characteristics of studies.	Yes
Synthesis of results	8	Present results for main outcomes, preferably indicating the number of included studies and participants for each. If meta-analysis was done, report the summary estimate and confidence/credible interval. If comparing groups, indicate the direction of the effect (i.e. which group is favoured).	Yes
**DISCUSSION**			
Limitations of evidence	9	Provide a brief summary of the limitations of the evidence included in the review (e.g. study risk of bias, inconsistency and imprecision).	Yes
Interpretation	10	Provide a general interpretation of the results and important implications.	Yes
**OTHER**			
Funding	11	Specify the primary source of funding for the review.	
Registration	12	Provide the register name and registration number.	

24. Lupei, M. I. *et al.* A 12-hospital prospective evaluation of a clinical decision support prognostic algorithm based on logistic regression as a form of machine learning to facilitate decision making for patients with suspected COVID-19. *PLoS ONE* **17**, e0262193 (2022).
25. Young, A. J. *et al.* Using Machine Learning to Make Predictions in Patients Who Fall. *Journal of Surgical Research* **257**, 118–127 (2021).
26. Rodríguez, A., Mendoza, D., Ascuntar, J. & Jaimes, F. Supervised classification techniques for prediction of mortality in adult patients with sepsis. *The American Journal of Emergency Medicine* **45**, 392–397 (2021).
27. McCann-Pineo, M. *et al.* Predictors of emergency department opioid administration and prescribing: A machine learning approach. *The American Journal of Emergency Medicine* **46**, 217–224 (2021).
28. Jiang, H. *et al.* Machine learning-based models to support decision-making in emergency department triage for patients with suspected cardiovascular disease. *International Journal of Medical Informatics* **145**, 104326 (2021).
29. Zhu, F. *et al.* Machine learning models predict coagulopathy in spontaneous intracerebral hemorrhage patients in ER. *CNS Neurosci Ther* **27**, 92–100 (2020).
30. Ivanov, O. *et al.* Improving ED Emergency Severity Index Acuity Assignment Using Machine Learning and Clinical Natural Language Processing. *Journal of Emergency Nursing* **47**, 265-278.e7 (2021).
31. Klang, E. *et al.* Predicting adult neuroscience intensive care unit admission from emergency department triage using a retrospective, tabular-free text machine learning approach. *Sci Rep* **11**, 1381 (2021).
32. van Doorn, W. P. T. M. *et al.* A comparison of machine learning models versus clinical evaluation for mortality prediction in patients with sepsis. *PLoS ONE* **16**, e0245157 (2021).

33. Bolourani, S. *et al.* A Machine Learning Prediction Model of Respiratory Failure Within 48 Hours of Patient Admission for COVID-19: Model Development and Validation. *J Med Internet Res* **23**, e24246 (2021).

34. Soltan, A. A. S. *et al.* Rapid triage for COVID-19 using routine clinical data for patients attending hospital: development and prospective validation of an artificial intelligence screening test. *The Lancet Digital Health* **3**, e78–e87 (2021).

35. Heldt, F. S. *et al.* Early risk assessment for COVID-19 patients from emergency department data using machine learning. *Sci Rep* **11**, 4200 (2021).

36. Rojas-Mendizabal, V., Castillo-Olea, C., Gómez-Siono, A. & Zuñiga, C. Assessment of Thoracic Pain Using Machine Learning: A Case Study from Baja California, Mexico. *International Journal of Environmental Research and Public Health* **18**, 2155 (2021).

37. Yu, L. *et al.* Machine learning methods to predict mechanical ventilation and mortality in patients with COVID-19. *PLOS ONE* **16**, e0249285 (2021).

38. Yun, H. *et al.* Enhancement in Performance of Septic Shock Prediction Using National Early Warning Score, Initial Triage Information, and Machine Learning Analysis. *The Journal of Emergency Medicine* **61**, 1–11 (2021).

39. Chou, E. H. *et al.* Clinical Features of Emergency Department Patients from Early COVID-19 Pandemic that Predict SARS-CoV-2 Infection: Machine-learning Approach. *West J Emerg Med* **22**, 244–251 (2021).

40. Liu, N. *et al.* Utilizing machine learning dimensionality reduction for risk stratification of chest pain patients in the emergency department. *BMC Medical Research Methodology* **21**, 74 (2021).

41. Tan, T.-H. *et al.* Predicting outcomes in older ED patients with influenza in real time using a big data-driven and machine learning approach to the hospital information system. *BMC Geriatr* **21**, 280 (2021).

42. Song, X., Liu, M., Waitman, L. R., Patel, A. & Simpson, S. Q. Clinical factors associated with rapid treatment of sepsis. *PLOS ONE* **16**, e0250923 (2021).

43. Fenn, A. *et al.* Development and Validation of Machine Learning Models to Predict Admission From Emergency Department to Inpatient and Intensive Care Units. *Annals of Emergency Medicine* **78**, 290–302 (2021).

44. De Hond, A. *et al.* Machine learning for developing a prediction model of hospital admission of emergency department patients: Hype or hope? *Int J Med Inform* **152**, 104496 (2021).

45. Sung, S.-F., Hung, L.-C. & Hu, Y.-H. Developing a stroke alert trigger for clinical decision support at emergency triage using machine learning. *International Journal of Medical Informatics* **152**, 104505 (2021).

46. Karlsson, A. *et al.* Predicting mortality among septic patients presenting to the emergency department–a cross sectional analysis using machine learning. *BMC Emergency Medicine* **21**, 84 (2021).

47. Nguyen, M. *et al.* Developing machine learning models to personalize care levels among emergency room patients for hospital admission. *Journal of the American Medical Informatics Association* **28**, 2423–2432 (2021).

48. Huo, D. *et al.* Large Vessel Occlusion Prediction in the Emergency Department with National Institutes of Health Stroke Scale Components: A Machine Learning Approach. *Journal of Stroke and Cerebrovascular Diseases* **30**, 106030 (2021).

49. Xie, F. *et al.* Development and Assessment of an Interpretable Machine Learning Triage Tool for Estimating Mortality After Emergency Admissions. *JAMA Netw Open* **4**, e2118467 (2021).

50. Li, C. *et al.* Machine learning based early mortality prediction in the emergency department. *International Journal of Medical Informatics* **155**, 104570 (2021).

51. Lee, J.-T., Hsieh, C.-C., Lin, C.-H., Lin, Y.-J. & Kao, C.-Y. Prediction of hospitalization using artificial intelligence for urgent patients in the emergency department. *Sci Rep* **11**, 19472 (2021).

52. Wu, T. T., Zheng, R. F., Lin, Z. Z., Gong, H. R. & Li, H. A machine learning model to predict critical care outcomes in patient with chest pain visiting the emergency department. *BMC Emergency Medicine* **21**, 112 (2021).

53. Cardosi, J. D., Shen, H., Groner, J. I., Armstrong, M. & Xiang, H. Machine learning for outcome predictions of patients with trauma during emergency department care. *BMJ Health Care Inform* **28**, e100407 (2021).

54. Garrafa, E. *et al.* Early prediction of in-hospital death of COVID-19 patients: a machine-learning model based on age, blood analyses, and chest x-ray score. *eLife* **10**, e70640 (2021).

55. Faisal, M. *et al.* A comparison of logistic regression models with alternative machine learning methods to predict the risk of in-hospital mortality in emergency medical admissions via external validation. *Health Informatics J* **26**, 34–44 (2020).

56. Jang, D.-H. *et al.* Developing neural network models for early detection of cardiac arrest in emergency department. *The American Journal of Emergency Medicine* **38**, 43–49 (2020).

57. Klang, E. *et al.* Promoting head CT exams in the emergency department triage using a machine learning model. *Neuroradiology* **62**, 153–160 (2020).

58. Klug, M. *et al.* A Gradient Boosting Machine Learning Model for Predicting Early Mortality in the Emergency Department Triage: Devising a Nine-Point Triage Score. *J Gen Intern Med* **35**, 220–227 (2020).

59. Kim, J. *et al.* Machine learning for prediction of septic shock at initial triage in emergency department. *Journal of Critical Care* **55**, 163–170 (2020).

60. Mao, H.-F. *et al.* A new risk stratification score for patients with suspected cardiac chest pain in emergency departments, based on machine learning. *Chin Med J (Engl)* **133**, 879–880 (2020).

61. Fernandes, M. *et al.* Predicting Intensive Care Unit admission among patients presenting to the emergency department using machine learning and natural language processing. *PLOS ONE* **15**, e0229331 (2020).

62. Fernandes, M. *et al.* Risk of mortality and cardiopulmonary arrest in critical patients presenting to the emergency department using machine learning and natural language processing. *PLOS ONE* **15**, e0230876 (2020).

63. Chen, C.-H. *et al.* Emergency department disposition prediction using a deep neural network with integrated clinical narratives and structured data. *International Journal of Medical Informatics* **139**, 104146 (2020).

64. Mowbray, F., Zargoush, M., Jones, A., de Wit, K. & Costa, A. Predicting hospital admission for older emergency department patients: Insights from machine learning. *International Journal of Medical Informatics* **140**, 104163 (2020).

65. Hermans, J. J. R. *et al.* Chest CT for triage during COVID-19 on the emergency department: myth or truth? *Emerg Radiol* **27**, 641–651 (2020).

66. Yaghmaei, E., Ehwerhemuepha, L., Feaster, W., Gibbs, D. & Rakovski, C. A multicenter mixed-effects model for inference and prediction of 72-h return visits to the emergency department for adult patients with trauma-related diagnoses. *Journal of Orthopaedic Surgery and Research* **15**, 331 (2020).

67. Zhang, P.-I. *et al.* Real-time AI prediction for major adverse cardiac events in emergency department patients with chest pain. *Scand J Trauma Resusc Emerg Med* **28**, 93 (2020).

68. Kim, K. H. *et al.* Emergency department routine data and the diagnosis of acute ischemic heart disease in patients with atypical chest pain. *PLOS ONE* **15**, e0241920 (2020).

69. Plante, T. B. *et al.* Development and External Validation of a Machine Learning Tool to Rule Out COVID-19 Among Adults in the Emergency Department Using Routine Blood Tests: A Large, Multicenter, Real-World Study. *Journal of Medical Internet Research* **22**, e24048 (2020).

70. Naemi, A., Schmidt, T., Mansourvar, M. & Wiil, U. K. Personalized Predictive Models for Identifying Clinical Deterioration Using LSTM in Emergency Departments. in *Studies in Health Technology and Informatics* (eds. Värri, A. et al.) (IOS Press, 2020). doi:10.3233/SHTI200713.

71. Zhai, Q. *et al.* Using machine learning tools to predict outcomes for emergency department intensive care unit patients. *Sci Rep* **10**, 20919 (2020).

72. Rendell, K., Koprinska, I., Kyme, A., Ebker-White, A. A. & Dinh, M. M. The Sydney Triage to Admission Risk Tool (START2) using machine learning techniques to support disposition decision-making. *Emergency Medicine Australasia* **31**, 429–435 (2019).
73. Goto, S. *et al.* Artificial intelligence to predict needs for urgent revascularization from 12-leads electrocardiography in emergency patients. *PLOS ONE* **14**, e0210103 (2019).
74. Chiew, C. J. *et al.* Heart rate variability based machine learning models for risk prediction of suspected sepsis patients in the emergency department. *Medicine* **98**, e14197 (2019).
75. Raita, Y. *et al.* Emergency department triage prediction of clinical outcomes using machine learning models. *Critical Care* **23**, 64 (2019).
76. Zhang, X. *et al.* Advanced diagnostic imaging utilization during emergency department visits in the United States: A predictive modeling study for emergency department triage. *PLOS ONE* **14**, e0214905 (2019).
77. Wu, C.-C. *et al.* An artificial intelligence approach to early predict non-ST-elevation myocardial infarction patients with chest pain. *Computer Methods and Programs in Biomedicine* **173**, 109–117 (2019).
78. Patterson, B. W. *et al.* Training and Interpreting Machine Learning Algorithms to Evaluate Fall Risk After Emergency Department Visits. *Med Care* **57**, 560–566 (2019).
79. Blom, M. C., Ashfaq, A., Sant'Anna, A., Anderson, P. D. & Lingman, M. Training machine learning models to predict 30-day mortality in patients discharged from the emergency department: a retrospective, population-based registry study. *BMJ Open* **9**, e028015 (2019).
80. Hunter-Zinck, H. S., Peck, J. S., Strout, T. D. & Gaehde, S. A. Predicting emergency department orders with multilabel machine learning techniques and simulating effects on length of stay. *Journal of the American Medical Informatics Association* **26**, 1427–1436 (2019).
81. Levin, S. *et al.* Machine-Learning-Based Electronic Triage More Accurately Differentiates Patients With Respect to Clinical Outcomes Compared With the Emergency Severity Index. *Annals of Emergency Medicine* **71**, 565-574.e2 (2018).

82. Kuo, P.-J. *et al.* Derivation and validation of different machine-learning models in mortality prediction of trauma in motorcycle riders: a cross-sectional retrospective study in southern Taiwan. *BMJ Open* **8**, e018252 (2018).

83. Mao, Q. *et al.* Multicentre validation of a sepsis prediction algorithm using only vital sign data in the emergency department, general ward and ICU. *BMJ Open* **8**, e017833 (2018).

84. Taylor, R. A., Melnick, E., Fleishman, W. & Venkatesh, A. The Impact of Risk Standardization on Variation in CT Use and Emergency Physician Profiling. *American Journal of Roentgenology* **211**, 392–399 (2018).

85. Hong, W. S., Haimovich, A. D. & Taylor, R. A. Predicting hospital admission at emergency department triage using machine learning. *PLOS ONE* **13**, e0201016 (2018).

86. Kwon, J. *et al.* Validation of deep-learning-based triage and acuity score using a large national dataset. *PLOS ONE* **13**, e0205836 (2018).

87. Dusenberry, M. W., Brown, C. K. & Brewer, K. L. Artificial neural networks: Predicting head CT findings in elderly patients presenting with minor head injury after a fall. *The American Journal of Emergency Medicine* **35**, 260–267 (2017).

88. Horng, S. *et al.* Creating an automated trigger for sepsis clinical decision support at emergency department triage using machine learning. *PLOS ONE* **12**, e0174708 (2017).

89. Zhang, X. *et al.* Prediction of Emergency Department Hospital Admission Based on Natural Language Processing and Neural Networks. *Methods Inf Med* **56**, 377–389 (2017).

90. Taylor, R. A. *et al.* Prediction of In-hospital Mortality in Emergency Department Patients With Sepsis: A Local Big Data–Driven, Machine Learning Approach. *Academic Emergency Medicine* **23**, 269–278 (2016).

91. Zlotnik, A., Alfaro, M. C., Pérez, M. C. P., Gallardo-Antolín, A. & Martínez, J. M. M. Building a Decision Support System for Inpatient Admission Prediction With the Manchester Triage System and Administrative Check-in Variables. *CIN: Computers, Informatics, Nursing* **34**, 224–230 (2016).

92. Handly, N., Thompson, D. A., Li, J., Chuirazzi, D. M. & Venkat, A. Evaluation of a hospital admission prediction model adding coded chief complaint data using neural network methodology: *European Journal of Emergency Medicine* **22**, 87–91 (2015).

93. Jenny, M. A. *et al.* Are Mortality and Acute Morbidity in Patients Presenting With Nonspecific Complaints Predictable Using Routine Variables? *Academic Emergency Medicine* **22**, 1155–1163 (2015).

94. Liu, N. *et al.* Prediction of adverse cardiac events in emergency department patients with chest pain using machine learning for variable selection. *BMC Med Inform Decis Mak* **14**, 75 (2014).
